# Lifing Assessment of Gas Turbine Blade Root Affected by Out-of-Tolerances

**DOI:** 10.3390/ma17194881

**Published:** 2024-10-04

**Authors:** Federico Manzini, Alessandra Cesaretti, Andrea Bessone, Francesco Bagnera, Daniele Botto

**Affiliations:** 1 Department of Mechanical and Aerospace Engineering, Politecnico di Torino, 10129 Torino, Italy; federico.manzini@polito.it (F.M.); alessandra.cesaretti@polito.it (A.C.); 2 Ansaldo Energia, 16152 Genova, Italy; andrea.bessone@ansaldoenergia.com (A.B.); francesco.bagnera@ansaldoenergia.com (F.B.)

**Keywords:** fatigue, blade, fir-tree attachment, out-of-tolerance, lifing

## Abstract

Current and future heavy-duty gas turbines (GTs) are being developed as an alternative or support to renewable energy sources (RESs). Therefore, GTs are subjected to several instances of being switched on and off; thus, the material fatigue limit can be reached in a short time. In such a scenario, possible out-of-tolerances (OoTs) in critical components must be considered. In this paper, OoTs related to critical parameters in the attachment geometry of a rotor blade are considered to estimate their impact on component life through a 2D finite element (FE) analysis. First, a mesh refinement is performed to obtain mesh-independent results; second, OoT geometries are simulated to determine stresses and strains at the blade attachment and disc groove. The mesh refinement process is critical to ensure model accuracy for both nominal and OoT geometries. The results show that OoTs can lead to important reductions in the life of the intended component, both on the blade and disc sides. These results could be useful in updating the maintenance plan for components and could be used for future insights, with further work extending the study to 3D geometry, for example, and evaluating the effect of other geometries.

## 1. Introduction

### 1.1. Background and Motivation

In recent decades, the world has faced global warming, and this has led to new environmental challenges from the point of view of power generation, with increasing employment of renewable energy sources (RESs). These new challenges may involve, for example, increasing efficiency or reducing CO_2_ and NO_x_ emissions, but also understanding how to efficiently couple GTs with RESs. In this scenario, gas turbines (GTs) are seen as a possible support for RESs when they cannot meet energy requests. The new operating conditions involve numerous start-ups, shutdowns, and off-design conditions that can quickly lead to end of life. Moreover, increasingly stringent emission requirements have made it necessary to improve the efficiency and performance of GTs.

New design solutions are pushing GT components closer and closer to the structural limits of the material. Among these components, turbine blades and their attachments are certainly those that undergo the most demanding operating conditions due to high centrifugal loads and temperatures.

The machining process for the turbine blade attachment is a critical step in the manufacturing of the blade, considering that the tolerances are very tight, to ensure the structural safety of the component. Failure to meet these tolerances alters the nominal stress state and, in the worst cases, increases the equivalent stress. This increases the damage under cyclic loading and reduces the component life, leading to unexpected failure [1,2,3].

According to [4], blade attachment failure is mainly caused by four phenomena: high cycle fatigue (HCF), which causes failure in areas with localized stress concentrations; low cycle fatigue (LCF), caused by subsequent switching on and off; fretting fatigue, a phenomenon that can lead to failure because of the combined action of micro-slip in contact surfaces and high loads (e.g., centrifugal force); and creep, which causes permanent deformations due to long-term exposure to high temperature and high stress even if it is below the yield strength of the material.

Recent works have investigated the possibility of finding the optimum blade attachment geometry by employing optimization algorithms such as genetic algorithms (GAs). Single- and multiple-objective optimizations were performed to try to find an optimal geometry. In [5], the authors attempted to minimize the von Mises equivalent stress (single-objective) in a dovetail geometry; the same stress was optimized in fir-tree geometry in [5]. The work presented in [6] focused on finding a combination of a set of parameters that minimized stress distributions in terms of von Mises stress, principal stresses, and contact pressures, thus minimizing the impact of fretting fatigue on a blade fir-tree attachment. This work is an example of multi-objective optimization, in which stresses (von Mises, principal, and shear stress) and contact pressure are minimized, first together and then one-by-one, to find the best optimum among different geometries. Fretting wear [7] is also a non-negligible phenomenon in blade attachments as a source of failure. The work presented in [8] shows that a disc slot with a mounting stagger angle to the axial direction introduced an increase in contact pressures and slip amplitude up to 100% compared with a straight slot, and this can affect fretting. As explained in [9], to ensure safe stress values on the blade–disc joint region, a suitable geometry must be designed. Moreover, as reported in [10], stresses in the contact region can be challenging to bring to convergence due to non-linearities related to Coulomb friction laws. Hence, it is important to verify the stress convergence using a mesh refinement process.

In [11], attention was focused on the disc fir-tree attachment, and critical geometry features were taken into account, such as the number of teeth, the contact angle, and the skew angle. A mechanical integrity verification on a steam turbine blade–disc T-root was carried out in [12] to develop design rules for the validation of these blades. Finite element (FE) analysis was performed as a design check.

The work presented in [13] aimed to minimize the mass of a fir-tree attachment. Another work dealing with single-objective optimization is presented in [14], where the minimized function was the peak stress in the notch region. In [15], the authors tried to achieve the minimum plastic strain. The optimizations reported in [16] are both single-objective and multi-objective: single-objective optimizations were performed to minimize the mean contact pressure on a dovetail geometry and the von Mises equivalent stress on a fir-tree geometry; multi-objective optimization was performed on a dovetail geometry to minimize the first principal stress and the mean contact pressure. The work presented in [17] deals with the minimization of contact pressure on a dovetail attachment. This analysis requires a huge computational cost, so authors have tried different methods to reduce the convergence time. An adopted solution involves embedding metamodels into the GA, also called surrogated models, to reduce the number of call-backs in a high-fidelity model while the optimization algorithm is running. Metamodels are built using design of experiments (DOE) points. Another possibility is introducing a penalty method to better manage non-feasible solutions before running the optimization algorithm. An in-house analytical tool based on a 1D approach to the conceptual design phase that excludes non-feasible solutions from the searching domain was developed in [5]. All of the works cited are limited to finding the optimal geometry of the attachment and do not provide guidance on manufactory tolerances.

A similar analysis was carried out in [18], where the focus was put on the disc geometry. The optimization algorithm was multiple-objective, and the selected objectives involved the stresses in some critical spots. The aim was to achieve a minimum weight configuration.

The impact of design tolerances on blade fir-tree attachments was analyzed in [19], and the results showed a reduction in component life.

In the last few years, new blade fir-tree attachments with barrel contacts have been proposed as having the potential to reduce stresses on contacts. The results show that curved contacts decrease peak pressures on contacts [20] and that the life of the attachment is longer. In the same work, the effect of manufactory tolerances on blade attachments was considered, and the results showed a 60 percent increment in Von Mises peak stress.

### 1.2. Objective

This work aims to thoroughly investigate the effect of the out-of-tolerance (OoT) machining on the fatigue life of blade attachments. To the authors’ knowledge, there are no papers in the literature where such an analysis has been carried out. Then, for the first time, the state of stress in the blade attachment has been evaluated under OoT conditions. In addition, this analysis can help the company better manage nonconformities: a component affected by OoT may be able to function for a shorter period; therefore, it is not discarded out of hand.

This paper is organized as follows. Section 2 describes, in detail, the steps followed to achieve the prefixed goal, while Section 3 shows the 2D model of the disk and blade attachment. The FE model is illustrated in Section 4, which also describes the mesh refinement process, performed to achieve mesh-independent results, and the setting of the contacts elements, which are a critical aspect of the study. In Section 5, a brief description of the tool used for the LCF assessment is given; Section 6 reports nominal geometry results, while Section 7 describes the stress gradient approach used as an alternative method to evaluate the fatigue life of the blade fir-tree attachment; Section 8 shows the statistical analysis on the OoT values to define which parameters were more affected or relevant for the analysis. Section 9 reports the most-representative OoT results and compares the results of nominal geometries and OoT to quantify their impact on component life. Section 10 contains the conclusions and closes the paper.

## 2. Procedures and Methods

**Figure 1:**
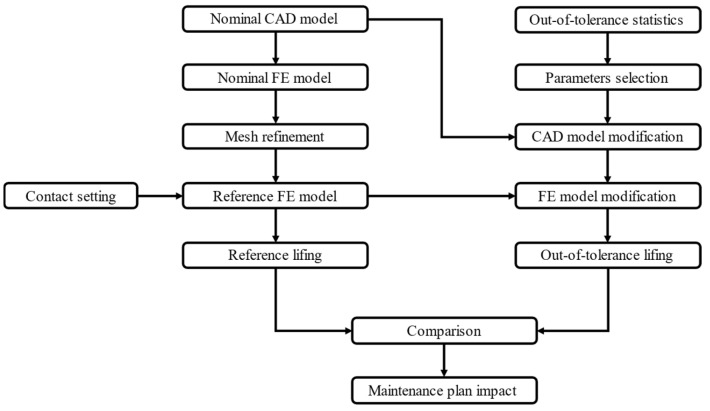
Procedure flowchart.

To achieve the set objective, a GT turbine blade with a three-lobed fir-tree attachment was chosen as a case study. The fir-tree attachment was modeled with a simplified two-dimensional (2D) sub-model. Details of the blade geometry are provided in Section 3. A consistent procedure was established to thoroughly evaluate the impact of the OoT geometries in the fir-tree attachment. A schematic flowchart is reported in Figure 1.

The first step is to generate the 2D nominal geometry model by cutting a section of the 3D solid model of the blade. In the next step, the 2D nominal model is imported into ANSYS to generate the FE model, along with material data and the thermal and mechanical boundary conditions (discussed in detail in Section 4.1). The blade and the disc are made of a nickel-based superalloy [21] and martensitic steel [22], respectively.

To obtain mesh-independent results, a mesh refinement process was performed. Refinement is an iterative process that reduces the mesh size near the contact at each iteration to evaluate the effect of the mesh size on the contact solution. In the meantime, different settings were tried out to best represent the contact, such as the contact’s behavior, detection point, initial penetration, and solving algorithm. The optimal choices were incorporated into the reference FE model (which will be described more precisely in the next sections). LCF life estimation was performed for nominal geometry using this model. The next step was to study the effect on component life, considering the OoT geometries of the attachment. A statistical analysis was carried out to find out which parameters were most affected by OoT. Based on the previous analysis, a parametric 2D model was prepared to easily modify the geometry. The modified geometries were then imported into ANSYS to create the OoT FE models. Finally, an LCF calculation was performed on the OoT geometries and then was compared with the nominal and minimum component requirements. The comparison is critical in deciding whether or not the maintenance program should be revised.

## 3. Two-Dimensional Model

**Figure 2:**
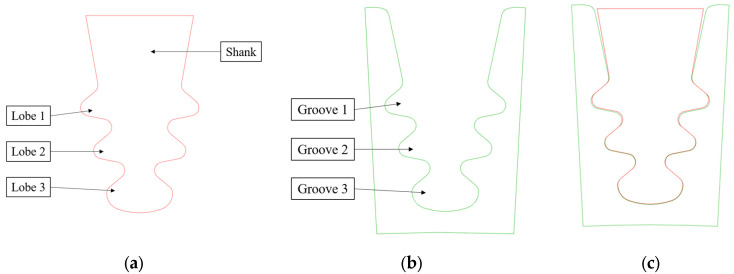
(**a**) 2D representation of the fir-tree attachment; (**b**) 2D representation of the disc section; (**c**) CAD model with nominal geometry.

Considering that the reported OoTs were related to the blade fir-tree attachment, an upper cut of the 3D model of the blade was made to remove the airfoil, and a lower cut of the 3D model of the disc was made a few millimeters below the disc groove. The reference section is the mid-section with respect to the axial direction. Figure 2 shows the 2D model used for the analysis.

## 4. FE Model Settings

The FE model was developed in ANSYS Workbench (2019 R2) and ANSYS Mechanical APDL (2019 R2), performing a 2D Static Structural analysis. A plane strain state with a constant (but non-zero) strain along the axial direction was assumed to allow for thermal expansion. Two-dimensional elements with eight nodes (PLANE183) were used for the blade attachment and disc groove, while the contact elements were one-dimensional three-noded CONTA172 and the target elements were one-dimensional three-noded TARGE169 [23]. The PLANE183 nodes were chosen because they are second-order elements and provide more accurate results for complex geometries. Some compatible contact elements are CONTA172 and TARGE169.

### 4.1. Boundary Conditions

In the lower cut section, a displacement computed with a 3D FE analysis of the same blade was imposed. A constant force was applied in the top cut section to simulate the centrifugal force of the blade mass above the cut. Cooling channels inside the blade were not modelled in this analysis. Bending effects can be neglected because the blade’s center of mass is designed to balance the bending effect due to hot gas pressures on the airfoil (at least at nominal loading conditions). Since the model is symmetrical with respect to the radial direction, zero hoop displacements are imposed on the axis of symmetry for both the disk and the blade.

### 4.2. Contact Settings

The main challenge when dealing with contact problems is setting the contact parameters to achieve reliable results. Therefore, the contact parameters were changed as shown below.

**Figure 3:**
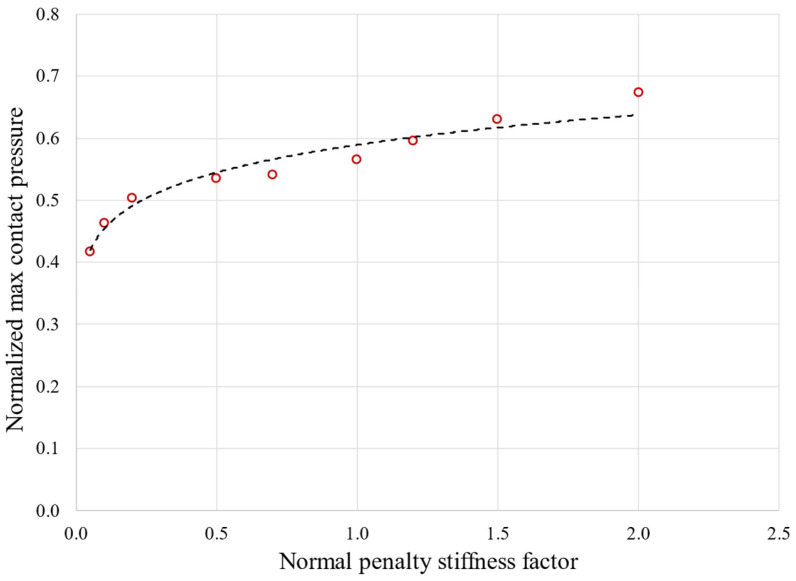
Effect of normal penalty stiffness factor on maximum contact pressure obtained with elastic calculation. Pressures are normalized using a reference value.

**Figure 4:**
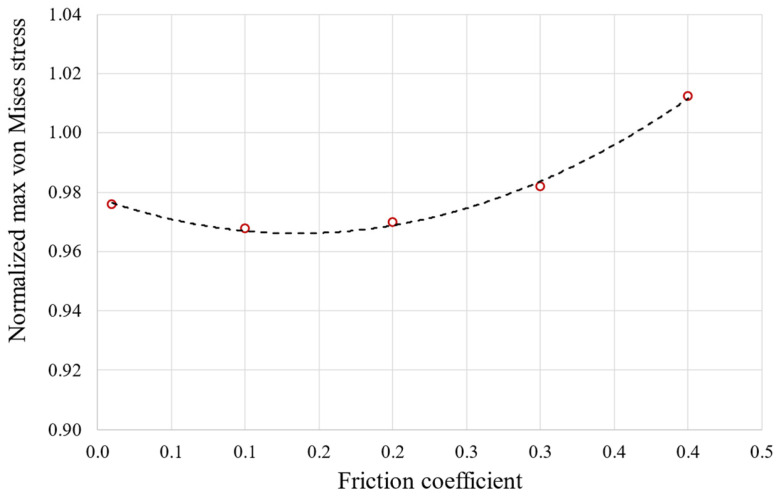
The effect of the friction coefficient on von Mises maximum stress obtained using elastoplastic calculation. The stress values are normalized with the yield stress of the material.

- Contact algorithm: set to Augmented Lagrange Method.
- Contact distribution on geometry: considering that contact could occur beyond the flat portion of the active plane, adjacent rounded edges were also added to the potential contact region.
- Normal penalty stiffness factor: manage penetration between contact and target (higher stiffness values decrease the amount of penetration [23]). Different values were tested (0.05, 0.1, 0.2, 0.5, 0.7, 1, 1.2, 1.5, and 2) and then the penalty stiffness factor was set to 1. Figure 3 shows the effect of the normal penalty stiffness factor effect on the normalized maximum contact pressure.

- Contact detection: can be set at Gauss points or at nodes. For the latter option, there are three possibilities: on nodes normal to the target, where the normal to the contact is perpendicular to the target surface; on nodes normal to the contact, where the normal to the contact is perpendicular to the contact surface; and the surface projection method, where the contact detection remains on the contact nodes, but the contact constraint is forced onto an overlapping region of the contact and target’s surfaces. The contact penetration/gap is computed over the overlapping region [23]. The chosen option is contact detection at Gauss points.
- Behavior of contact surface: options are standard or rough. For this present analysis, the option was set to standard.
- Sliding behavior: options are small or finite sliding. The behavior was set to finite sliding to ensure the convergence of the analysis was due to the very small size of the elements. The definition of small sliding is related to the size of the element.
- Initial penetration: include or exclude everything. Set to include everything to account for any gaps or penetrations.
- Automated contact adjustment: options are no automated adjustment, close gap, reduce penetration, close gap/reduce penetration, and default ICONT. This option was set to close gap/reduce penetration because it helps the convergence of the analysis to restore gaps or penetration at the first step of the analysis.
- Friction coefficient. Five values, namely 0.01, 0.1, 0.2, 0.3, and 0.4, were tested from which the von Mises stress was calculated as a function of the friction coefficient, as shown in Figure 4. The friction coefficient was then set to 0.2, a value commonly used in industrial applications.

### 4.3. Mesh Refinement Process

The objective of the mesh refining process is to achieve mesh-independent results. The sizes of the elements during the mesh refinement process are reported in Table 1.

**Figure 5:**
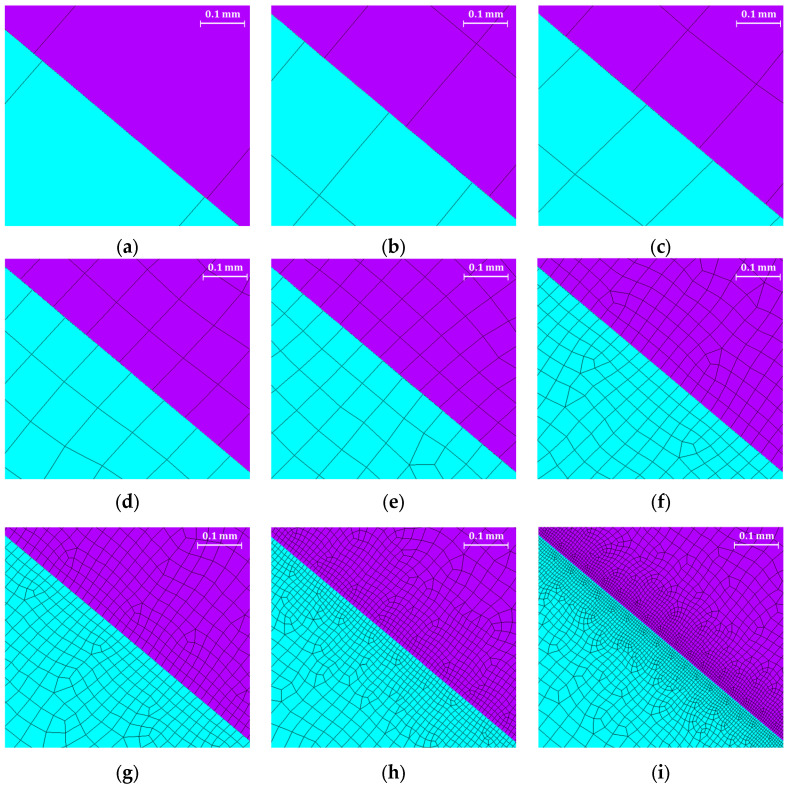
Comparison of mesh sizes. Contact sizes are as follows: (**a**) 0.5 mm; (**b**) 0.25 mm; (**c**) 0.2 mm; (**d**) 0.1 mm; (**e**) 0.06 mm; (**f**) 0.03 mm; (**g**) 0.02 mm; (**h**) 0.01 mm; (**i**) 0.005 mm. The different mesh colors, cyan and purple, identify the blade lobe and the disk groove, respectively.

**Figure 6:**
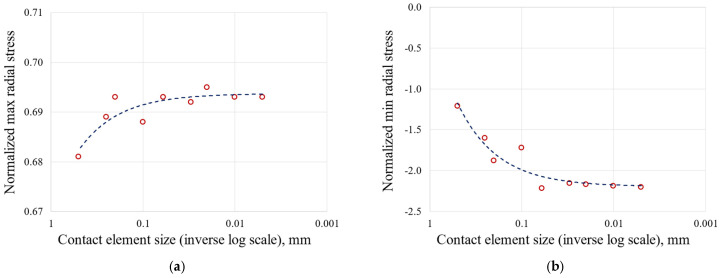

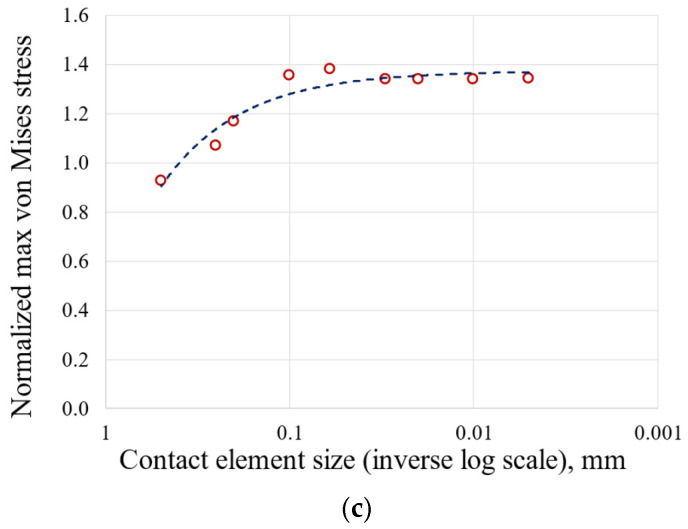
Stress as a function of contact element size on the blade attachment: (**a**) maximum radial stress; (**b**) minimum radial stress; (**c**) maximum von Mises stress.

**Figure 7:**
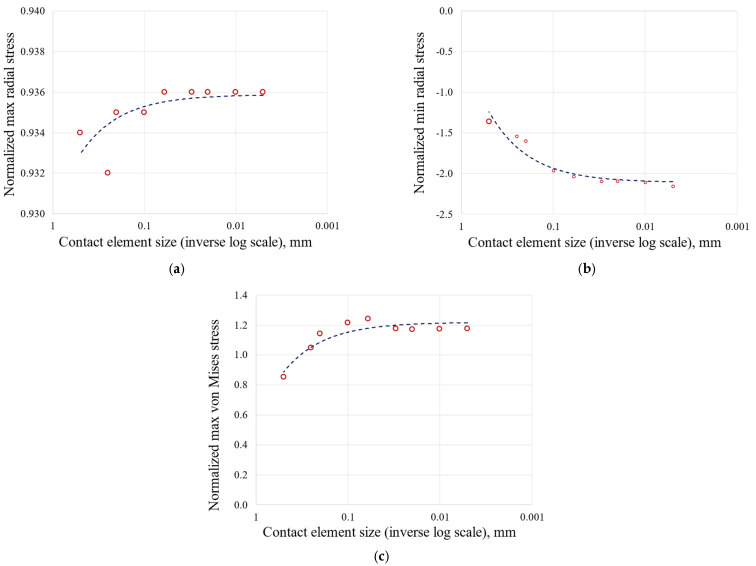
Stress as a function of contact element size on the disc groove: (**a**) maximum radial stress; (**b**) minimum radial stress; (**c**) maximum von Mises stress.

**Figure 8:**
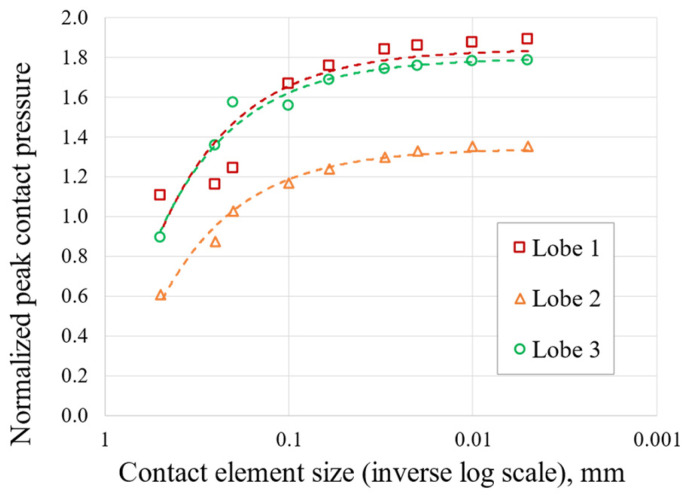
Trend in contact pressure versus contact element size.

**Figure 9:**
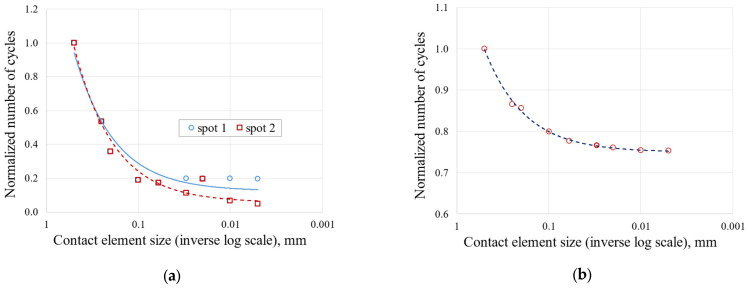
Effect of contact mesh size on the normalized number of cycles to crack initiation: (**a**) blade attachment; (**b**) disc groove.

The 2D approach and the symmetry assumption are key to achieving this level of refinement. It would have been unthinkable to perform a 3D FEA on the entire blade and the correspondent disc sector with elements small enough to achieve a stable stress state. To compare the meshes, an equal zoom is made for all of them and is shown in Figure 5. Table 2, Table 3 and Table 4 and Figure 6, Figure 7, Figure 8 and Figure 9 show asymptotic stress trends when the mesh size is finer, so it can be said that these results are mesh-independent.

**Figure 10:**
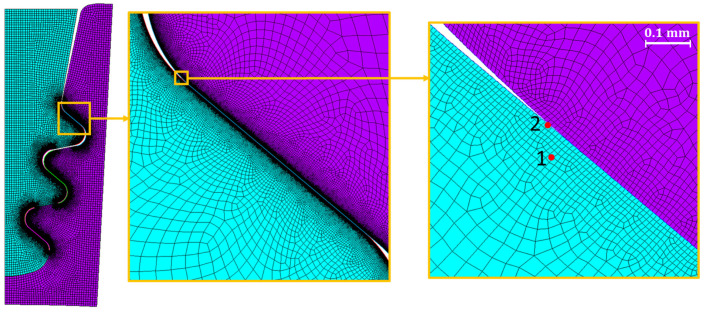
Mesh selected for analysis.

Figure 9 shows the trend in the number of cycles to crack initiation versus mesh size for both the blade fir-tree (Figure 9a) and the disc groove (Figure 9b). For the convergence of the number of cycles of the blade, two critical spots were considered. These spots are indicated with two red circles in Figure 10. As shown in Figure 6, Figure 7, Figure 8 and Figure 9, the mesh that ensures mesh-independent results and acceptable computational cost is the one named 693k, that is, the one with contact element size of 0.01 mm. The final mesh consists of 692,708 nodes and 242,124 elements. An image of the selected mesh is shown in Figure 10.

## 5. AEN In-House Tools for LCF Evaluation

### 5.1. Tool for Blade Fir-Tree Attachment LCF Evaluation

**Figure 11:**
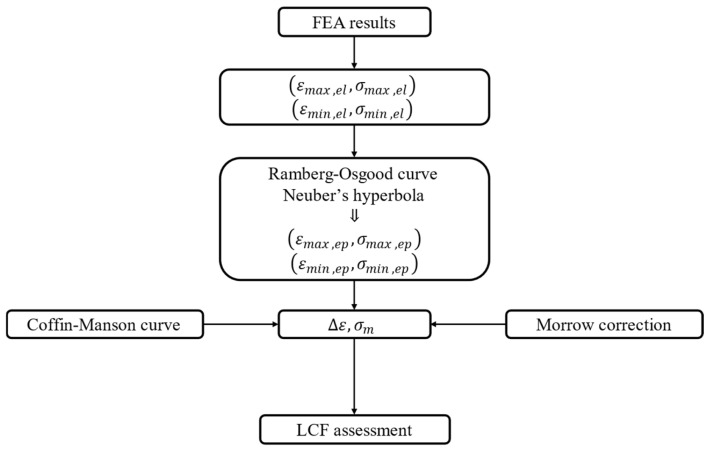
Procedure for estimating the fatigue life of blade attachments.

To evaluate the stresses and number of cycles under LCF conditions, it is necessary to carry out an elastic analysis by considering at least two-time steps. The duty cycle considered for the analysis consists of a transitory from start to Base Load (BL). The in-house tool implements a procedure shown in Figure 11, according to the theory explained in [24] and following the recommendation provided in [2,3].

### 5.2. Tool for Rotor Disc LCF Evaluation

**Figure 12:**
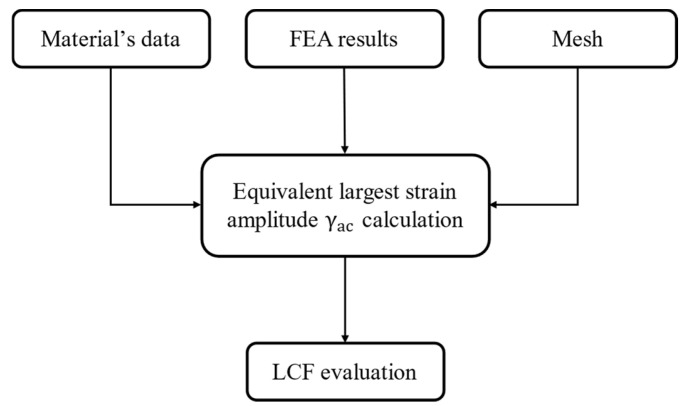
Procedure for estimating the fatigue life of disc grooves. Nominal geometry results.

The tool evaluates multiaxial fatigue from the elastic–plastic calculation, and uses Socie’s criterion to evaluate the maximum amplitude of the shear strain in the critical plane.

(1) $$\gamma_{ac}\left(1+\frac{\alpha \sigma_{max,c}}{\sigma_{o}^{’}}\right)=\frac{\tau_{f}^{’}}{G}{\left(2N_{f}\right)}^{b}+\gamma_{f}^{’}{\left(2N_{f}\right)}^{c}$$

where $\gamma_{ac}$ is the maximum amplitude of the shear strain in the critical plane [25]. A simplified scheme of the procedure implemented by the tool is reported in Figure 12.

## 6. Results Obtained on Nominal Geometry

Below are the most relevant results obtained with the nominal geometry that are taken as a reference for comparison. 

### 6.1. Stress Distribution

**Figure 13:**
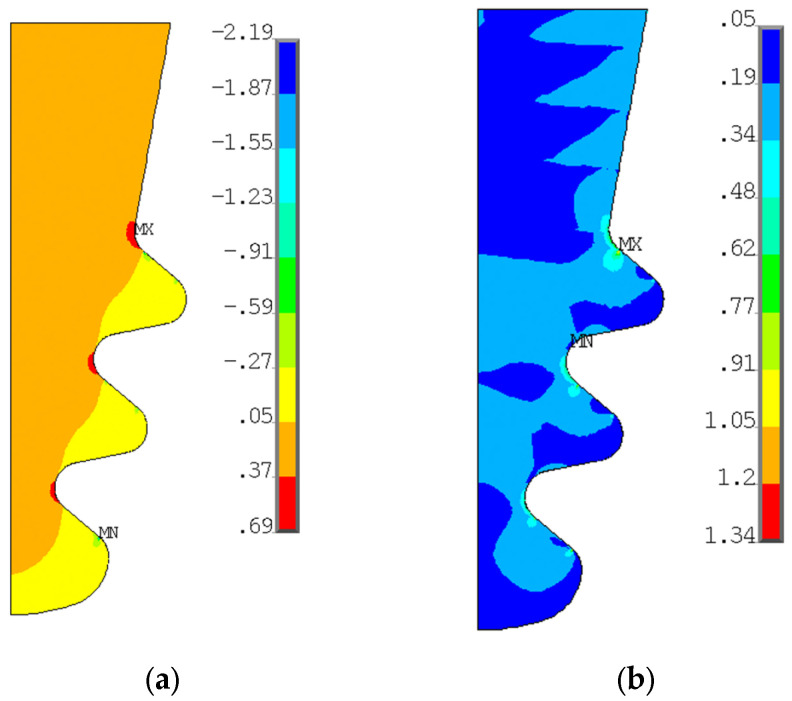
(**a**) Radial stress distribution on blade fir-tree attachment from elastic calculation; (**b**) von Mises stress distribution on blade fir-tree attachment from elastic calculation. Both stresses are normalized with a reference value.

**Figure 14:**
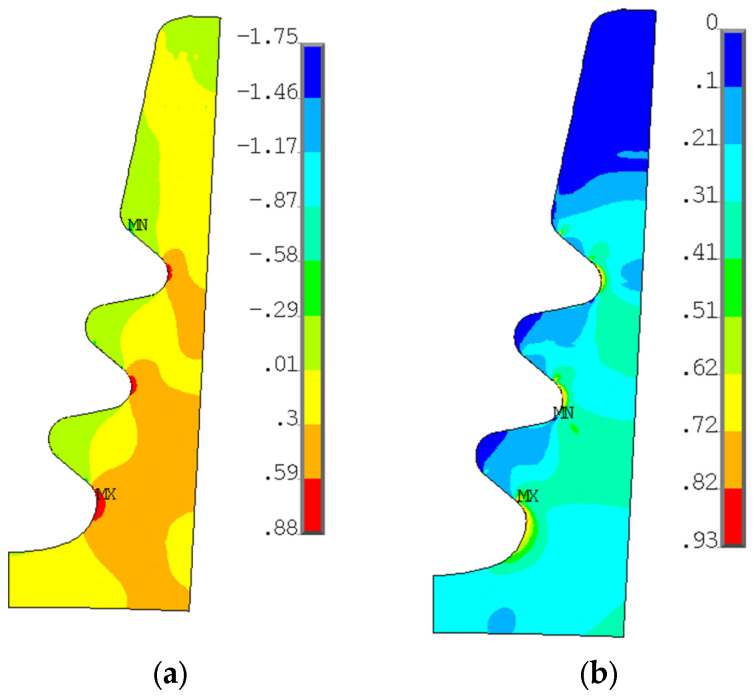
(**a**) Radial stress distribution on disc groove from elastic-plastic calculation; (**b**) von Mises stress distribution on the disc groove from the elastic–plastic calculation. Both stresses are normalized with a reference value.

**Figure 15:**
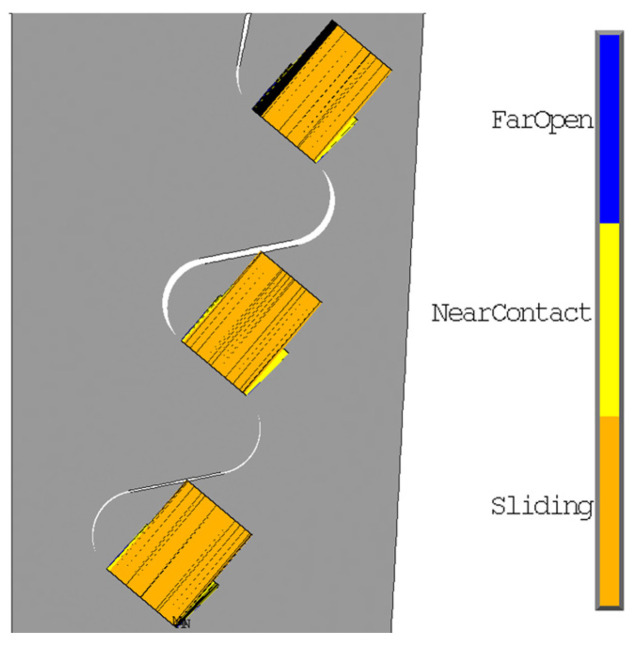
Contact status from elastic calculation.

**Figure 16:**
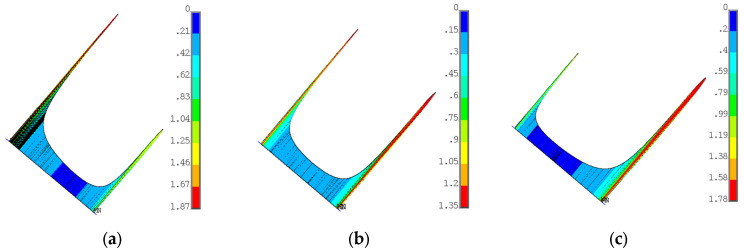
(**a**) Contact pressure on lobe 1 from elastic calculation; (**b**) contact pressure on lobe 2 from elastic calculation; (**c**) contact pressure on lobe 3 from elastic calculation. Pressures are normalized with a reference value.

Figure 13 shows the radial and von Mises stresses derived from an elastic calculation on the blade fir-tree. Figure 14 shows the same stresses from an elastoplastic analysis on the disc groove. Figure 15 and Figure 16 show the contact status and pressures.

Consistent with the expected results, stresses on the top part of both the attachment and the disc are almost zero. Moreover, the radial tensile stress is localized on inner curved edges. The tensile stress reports higher values on the disc than on the attachment. The active planes of the attachment report compressive stresses, with two small zones reporting very high values, the location of which corresponds to where the contact ends. In this position, there is also a high value of von Mises stress, because the disc is the same, but there are also the inner curved edges which report a high von Mises stress value.

As expected for flat contacts, there are two peaks at the end of the contacts. If the contact is rounded, these peaks are smaller [20]. The location of high compression zones corresponds to these peaks.

### 6.2. LCF Evaluation

**Figure 17:**
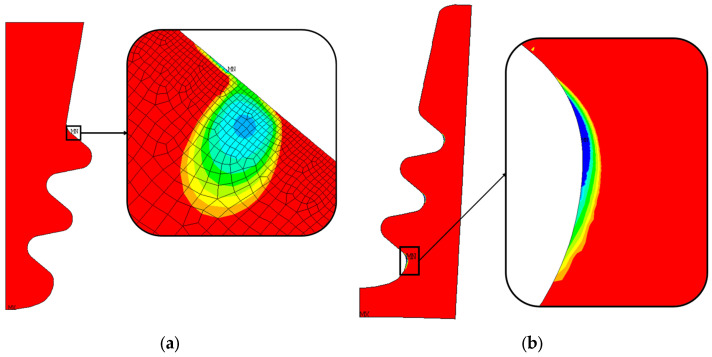
Distribution of the number of cycles to crack initiation and critical zone for nominal geometry. The number of cycles grows from blue to red; (**a**) blade fir-tree attachment, (**b**) disc groove.

Figure 17a shows the number of LCF cycles to crack initiation (the number of cycles grows from blue to red). As stated in Section 5.1, these results are obtained from an elastic calculation. Almost everywhere, the blade attachment exceeds the maximum considered LCF value. The value chosen as the reference is the minimum number of cycles, corresponding to the blue region. 

As for the blade attachment, and also for the disc groove, the number of cycles exceeds the maximum reference value (see Figure 17b). There are three main zones that report a lower number of cycles, and those zones are near the inner curved edge of the groove. Consistent with the stress distribution, these zones are those that report high radial tensile stress and high von Mises equivalent stress, as shown in Figure 14. These results are obtained from elastoplastic calculations.

## 7. Stress Gradient Approach

The local approach to blade fir attachment lifing applied by Ansaldo Energia and implemented in the in-house tool may be overly conservative, considering that there may be very localized stress concentrations. To account for this, a non-local approach based on the stress gradient [26,27] can be used, which should lead to an increase in the number of cycles. Obviously, if this approach leads to a lower number of cycles, it means that it cannot be applied, because the gradient is too low.

**Figure 18:**
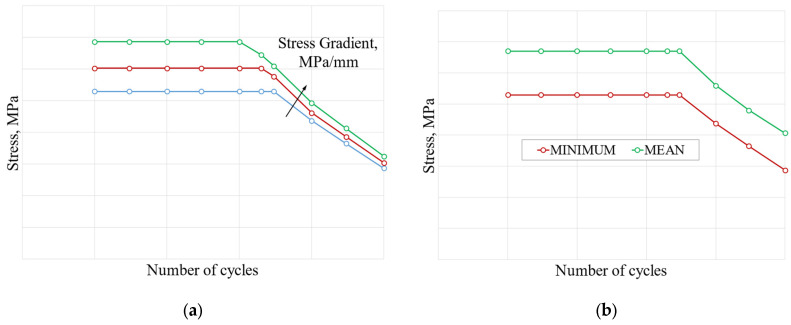
(**a**) S-N curve for different stress gradients; (**b**) mean and minimum S-N curve for the same stress gradient. (**a**) S-N curve for different stress gradients; (**b**) mean and minimum S-N curve for the same stress gradient.

The stress gradient approach is based on experimental tests on notched samples that provide several S-N curves parameterized by temperature and stress gradient value. Figure 18a shows an example of these curves. Figure 18b shows, for a given stress gradient, the design curve and mean curve.

The number of cycles can be predicted using the S-N curve if the stress gradient value is known. The stress gradient is evaluated by selecting a reference point and direction. Distance is calculated as

(2) $$d=\sqrt{{\left(x-x_{ref}\right)}^{2}+{\left(y-y_{ref}\right)}^{2}}$$

while the stress gradient is

(3) $$\nabla \sigma =\frac{\sigma -\sigma_{ref}}{d}$$

**Figure 19:**
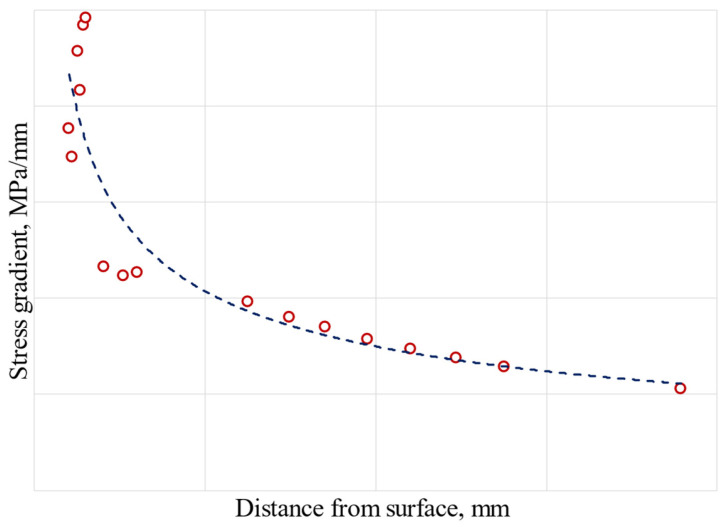
Nominal geometry–stress gradient evaluation.

In this analysis, the critical node from the perspective of LCF is taken as the reference to evaluate stress gradient. The von Mises stress gradient is calculated along the normal to active plane from the contact surface to a reference distance within the material. The stress gradient as a function of the reference distance for the nominal geometry is plotted in Figure 19 as an example.

For the nominal geometry, the ratio of the number of cycles between the two methods, local approach, and stress gradient is 3.65, which means a 265% increase using the stress gradient. The stress gradient curve used for the analysis was the design curve.

## 8. Out-of-Tolerance Statistics

**Figure 20:**
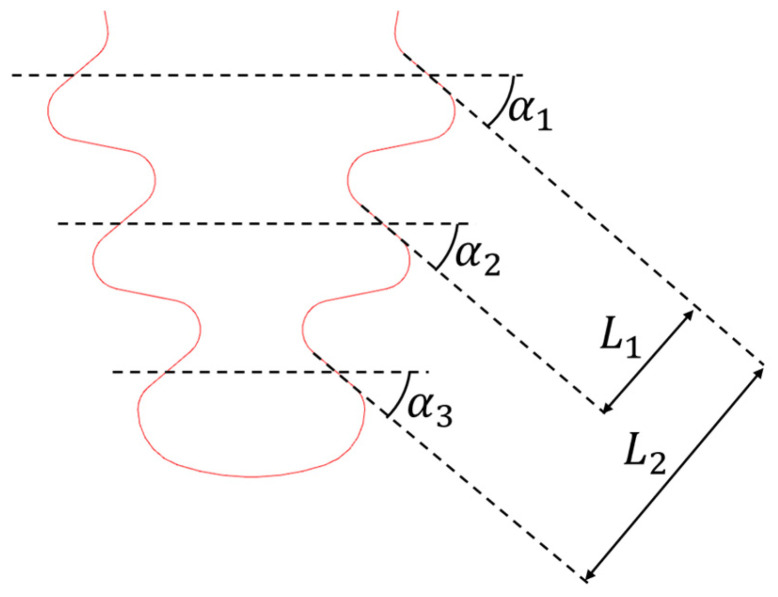
Parameters chosen for the analysis.

The blade fir-tree attachment has many parameters that can be affected by out-of-tolerances. Therefore, it was necessary to decide which ones were most relevant to the analysis and report more of the OoTs. The selected parameters are shown in Figure 20.

**Figure 21:**
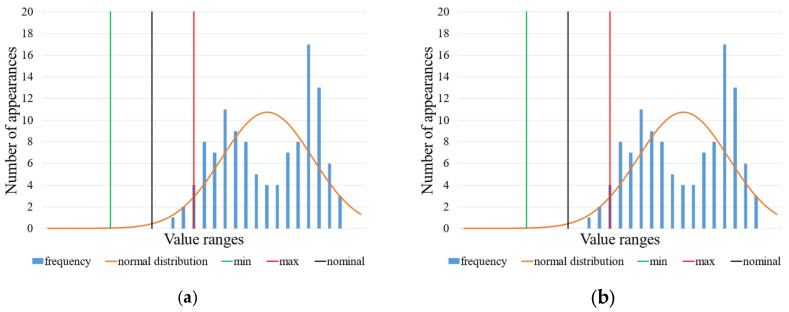

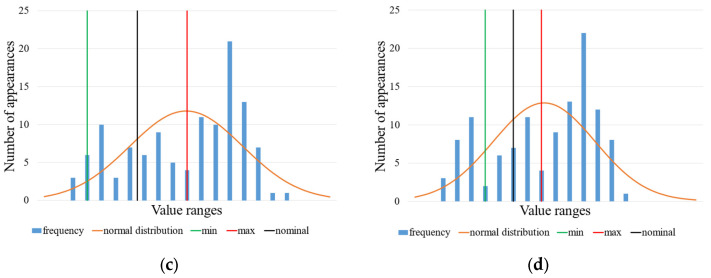
(**a**) Distribution of the out-of-tolerances in the active plane angle $\alpha_{2}$. Angle $\alpha_{1}$ was not found to be OoT, but is chosen because its mid-point is the reference for measuring the other parameters. (**b**) Distribution of the out-of-tolerance in the active plane angle $\alpha_{3}$. (**c**) Distribution of the out-of-tolerances in the active plane offset *L_1_*. (**d**) Distribution of the out-of-tolerances in the active plane offset *L_2_*. The black vertical line represents the nominal value of the parameter; the green (min) and red (max) vertical lines represent the lower limit and the upper limit of the tolerance range, respectively.

A statistical analysis of the data collected among some series of machined blades was performed assuming a normal distribution. Figure 21 shows the results of the statistical analysis for the selected parameters. In some cases, the normal distribution does not fit the statistical data correctly, so for this reason (but also to be as conservative as possible) the selected OoT values are the maximum measured values and not a statistic of the normal distribution.

**Figure 22:**
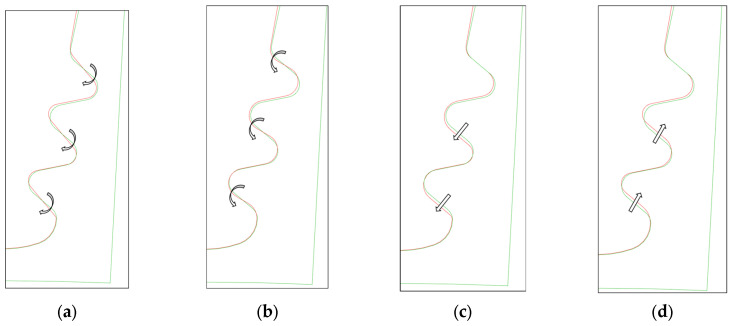
(**a**) Angle upper tolerance rotation; (**b**) angle lower tolerance rotation; (**c**) offset upper tolerance translation; (**d**) offset lower tolerance translation; (**a**,**c**) also represent the out-of-tolerance cases reported in Figure 21.

For sake of completeness, it can be noted that a certain error level needs to be considered in angles and offset measurements because of small contact surfaces (see Section 8.1). The OoT parameters are described below and are depicted in Figure 22.

- Angle lower tolerance: counterclockwise rotation in respect to the active plane mid-point.
- Angle upper tolerance: clockwise rotation in respect to the active plane mid-point.
- Offset lower tolerance: penetration between lobes.
- Offset upper tolerance: gap between lobes.
- Maximum out-of-tolerance: maximum value reported in out-of-tolerance statistics; variation is like upper tolerance.

### 8.1. Test Matrix

All possible combinations of parameters and non-nominal values would be too many to analyse, so only the most representative were considered. Table 5 reports the case studies analysed.

## 9. Results

Although several cases and combinations were tested, for the sake of compactness, this paper reports only the LCF evaluation results of the three most representative out-of-tolerance cases studies. The stresses and contact results are consistent with the changes applied to the fir-tree attachment geometry.

### 9.1. Third Active Plane Inclination Angle at Maximum Recorded Value in Out-of-Tolerance Statistics

**Figure 23:**
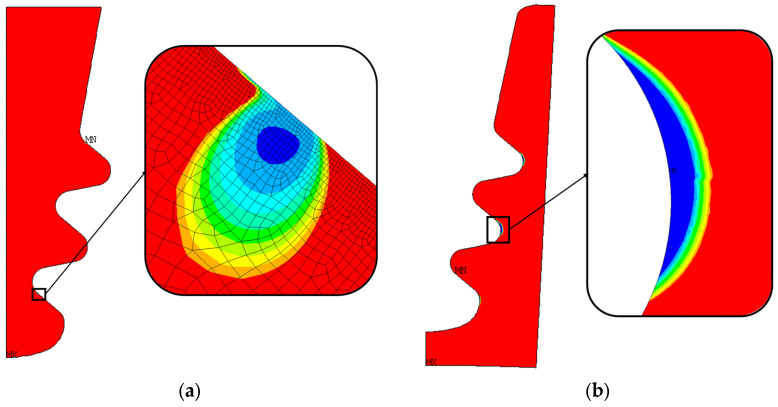
Distribution of the number of cycles to crack initiation and critical zone for out-of-tolerance geometry, case 9. The number of cycles grows from blue to red; (**a**) blade fir-tree attachment, (**b**) disc groove.

Figure 23 shows that almost everywhere the blade fir-tree attachment exceeds the minimum number of cycles considered as a target for LCF. The zone in which the minimum number of cycles is guaranteed is larger than in the nominal geometry calculation. Regarding the disc groove, the minimum number of cycles is on the second groove, whereas in the nominal case, it was on the third groove.

### 9.2. Second Active Plane Offset at Maximum Reported Value in Out-of-Tolerance Statistics

**Figure 24:**
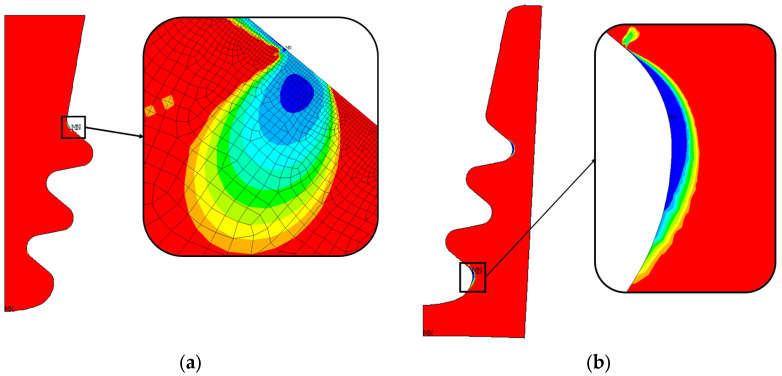
Distribution of the number of cycles to crack initiation and critical zone for out-of-tolerance geometry, case 24. The number of cycles grows from blue to red; (**a**) blade fir-tree attachment, (**b**) disc groove.

As shown in Figure 24, almost everywhere the blade fir-tree attachment exceeds the minimum number of cycles considered as a target for LCF. The zone with the minimum number of cycles is larger than the nominal geometry and includes the curved edge near the active plane. This means that, in this case, stresses here are greater than in the nominal geometry.

As for the disc, the minimum number of cycles is always on the third groove, but the blue zone is larger than the nominal geometry. There is also a larger green zone in the upper part of the figure.

### 9.3. All Parameters at Maximum Reported Value in Out-of-Tolerance Statistics

**Figure 25:**
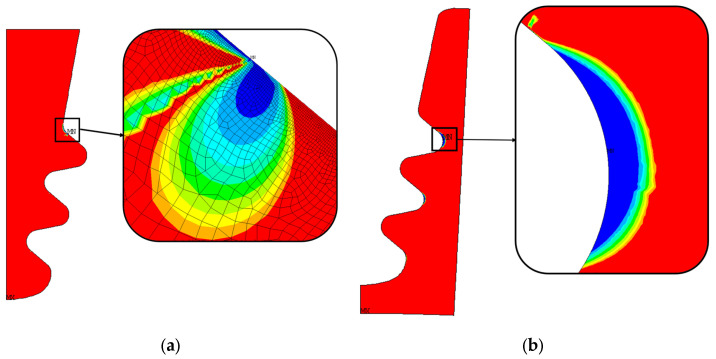
Distribution of the number of cycles to crack initiation and critical zone for out-of-tolerance geometry, case 30. The number of cycles grows from blue to red; (**a**) blade fir-tree attachment, (**b**) disc groove.

Also in this case (see Figure 25), almost everywhere the blade fir-tree attachment exceeds the minimum number of cycles considered as a target for LCF. The minimum number of cycle zones is larger than the nominal geometry and includes the curved edge near the active plane. This means that, in this case, stresses here are greater than in the nominal geometry.

In the disc, the minimum number of cycles occurs at the first groove. This is coherent with the new radial and von Mises stress distributions.

### 9.4. Comparison with Nominal Geometry

**Figure 26:**
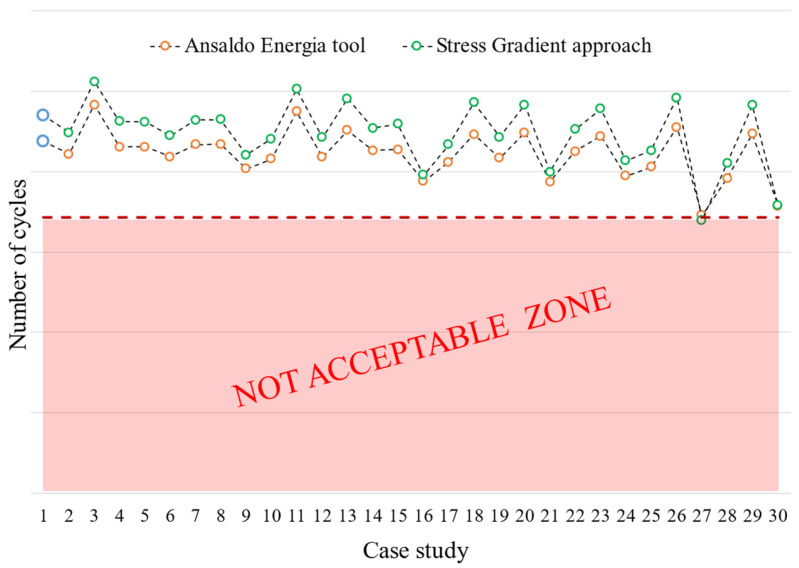
Blade attachment: Changes in the number of cycles (LCF) at crack initiation for all cases studied compared to the nominal geometry. The graph also shows a comparison between the local (AE in-house tool) and the gradient approach.

The results, in terms of the number of cycles to crack initiation, obtained from the evaluation of nominal geometry are taken as a reference for comparison. Figure 26 shows a case-by-case comparison of the LCF results. The results of the nominal geometry—case 1, the reference geometry—are indicated by blue circles.

All cases involving a counterclockwise rotation of the first active planes, i.e., the lower tolerance limit of angle $\alpha_{1}$ (cases number 3, 11, 13, 18, and 29), register an increase in the number of cycles. The same consideration can be applied to cases involving an upward shift in the active plane of lobes 2 and 3, i.e., the lower tolerance limit of offsets $L_{1}$ and $L_{2}$ (cases number 20, 23, 26, and 29). The increase in the number of LCF cycles is related to the fact that the LCF critical region (see Figure 17a) bears less load in these cases.

In terms of out-of-tolerance cases, it can be stated that a significant percentage of number of cycles is lost. The greatest reduction is recorded for cases number 27 ($L_{1,2}$ max) and 30 ($\alpha_{2,3}$, $L_{1,2}$ max), but cases number 16 ($\alpha_{2,3}$ max), 21 ($L_{1}$ max), and 24 ($L_{2}$ max) are also significant.

Other cases such as number 17 ($\alpha_{1,2,3}$ toll up), 25 ($L_{1,2}$ toll up), and 28 ($\alpha_{1,2,3}$, $L_{1,2}$ toll up) involve increased stresses and contact pressure in the critical region of the LCF (left side of the first active plane) and thus show a significant reduction in the number of cycles.

Finally, the two approaches to LCF estimation (AEN tool, orange circles, and stress gradient, green circles) give comparable results.

**Figure 27:**
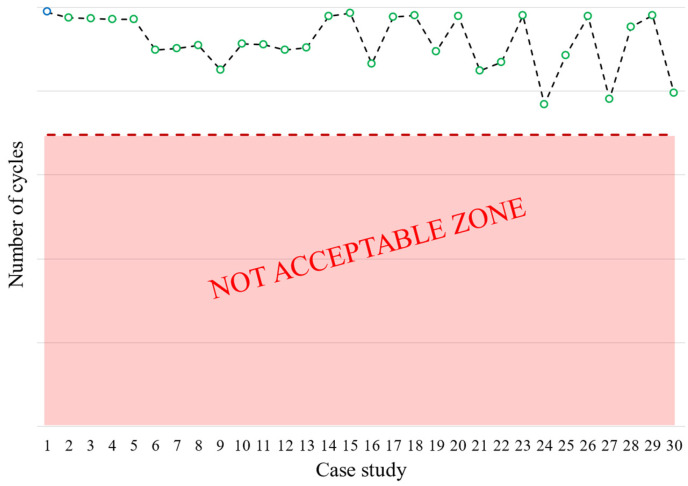
Disc groove: Changes in the number of cycles at crack initiation (LCF) for all of the cases studied compared to the nominal geometry.

As shown in Figure 27, any change in geometry causes a reduction in the fatigue life of the disk groove, and the most impactful cases are OoT. Cases number 24 ($L_{2}$ max), 27 ($L_{1,2}$ max), and 30 ($\alpha_{2,3},{\;L}_{1,2}$ max) record the largest losses, while case number 6 ($\alpha_{2}$ max) records the smallest reduction (among the OoT cases).

The critical region in nominal case (number 1) is the third groove (see Figure 17b). A significant reduction in fatigue life is also observed in cases number 19 ($L_{1}$ toll up), 22 ($L_{2}$ toll up), and 25 ($L_{1,2}$ toll up). The first case (19) involves a downward translation of the second active plane that causes an overload on grooves 1 and 3. The critical region remains on the third groove, and the number of cycles is reduced compared to the nominal geometry. The second case (22) considers a downward translation of the third active plane, causing the load on grooves 1 and 2 to increase and a critical region shift to the second groove. The last case (25) considers both the translation of the second and third active planes. The critical region shifts to the first groove because it now bears part of the load that was previously carried by grooves 2 and 3.

## 10. Conclusions

The results obtained from this activity led to the following conclusions.

- The statistical approach applied to the CTQ (Critical to Quality) measurements from the machining process permits us to better represent out-of-tolerances. In detail, this approach provides a more accurate method to quantify and evaluate the magnitude of the out-of-tolerances. Also, the trends in the machining process can be better investigated, and a final decision about which out-of-tolerance values deserve a more in-depth assessment can be made.
- Results from out-of-tolerance cases are consistent with the applied modifications to the nominal geometry, and the impact on the life of the blade attachment has been estimated.
- A relevant fatigue life decrement has been detected in several cases, especially when out-of-tolerances are considered.
- In addition, there are some case studies at the limits of tolerance that also show a non-negligible reduction in fatigue life. In all cases, there is a sufficiently large margin from the minimum number of cycles to crack initiation required from the product specification. On the other hand, the counterclockwise rotation of the active plane of the first lobe, i.e., $\alpha_{1}$ toll low, improves fatigue life on the blade root because it relieves the most-stressed point. Regardless, before a fatigue limit is reached, the blade can be disassembled, refurbished, and reconditioned.

All of the results have been obtained on an existing geometry for a component with a well-proven life duration. Therefore, the contents of this paper are useful for a comparison of calculation results and field results. Certainly, other parameters also need to be investigated with dedicated analyses, for example, the out-of-tolerance along the lobe in the axial direction (flatness of active planes). Furthermore, also in the case of a new blade design, these comparisons could be considered and applied to define a new tolerance range for a fir-tree attachment during the machining process.

Another goal achieved by this work is the establishment of an easy-to-implement procedure that can be applied to other similar components and geometries. A possible insight is to apply the same approach to similar fir-tree attachments and compare results to evaluate the impact of out-of-tolerances on the fatigue life of different turbine stages (with different centrifugal loads, materials, and temperatures).

Considering all of the above comments and possible insights, potential future goals could also include a critical review of the out-of-tolerance ranges, modifications of the component maintenance plans, or the establishment of an empirical formula as a function of the out-of-tolerance magnitude for the component lifing.

## Figures and Tables

**Table 1:** Dimensions of the mesh with different element sizes.

Mesh Name	Number of Nodes	Blade Surface Element Size [mm]	Disc Surface Element Size [mm]	Blade Contacts Edges Element Size [mm]	Disc Target Edges Element Size [mm]
70k	70,203	0.5	0.5	0.5	0.5
270k	276,696	0.25	0.25	0.25	0.25
43k	43,384	1	1	0.2	0.2
79k	78,557	1	1	0.1	0.1
125k	125,334	1	1	0.06	0.06
240k	241,340	1	1	0.03	0.03
355k	354,821	1	1	0.02	0.02
693k	692,708	1	1	0.01	0.01
1350k	1,350,673	1	1	0.005	0.005

**Table 2:** Blade attachment stresses. Results are normalized against a reference value.

Mesh Name	Maximum Radial Stress [-]	Minimum Radial Stress [-]	Maximum von Mises Stress [-]
70k	0.681	−1.211	0.927
270k	0.689	−1.604	1.069
43k	0.693	−1.878	1.169
79k	0.688	−1.723	1.357
125k	0.693	−2.217	1.382
240k	0.692	−2.154	1.342
355k	0.695	−2.171	1.340
693k	0.693	−2.189	1.340
1350k	0.693	−2.206	1.345

**Table 3:** Disc attachment stresses. Results are normalized against a reference value.

Mesh Name	Maximum Radial Stress [-]	Minimum Radial Stress [-]	Maximum von Mises Stress [-]
70k	0.934	−1.358	0.852
270k	0.932	−1.537	1.047
43k	0.935	−1.598	1.142
79k	0.935	−1.962	1.214
125k	0.936	−2.032	1.240
240k	0.936	−2.091	1.176
355k	0.936	−2.093	1.170
693k	0.936	−2.106	1.173
1350k	0.936	−2.152	1.177

**Table 4:** Comparison of the peak contact pressures. Results are normalized against a reference value.

Mesh Name	Lobe 1 Contact Pressure [-]	Lobe 2 Contact Pressure [-]	Lobe 3 Contact Pressure [-]
70k	1.104	0.608	0.894
270k	1.160	0.875	1.356
43k	1.245	1.029	1.571
79k	1.667	1.167	1.557
125k	1.758	1.241	1.685
240k	1.840	1.300	1.741
355k	1.859	1.329	1.756
693k	1.874	1.353	1.779
1350k	1.892	1.354	1.784

**Table 5:** Case studies.

Test	$\alpha_{1}$	$\alpha_{2}$	$\alpha_{3}$	$L_{1}$	$L_{2}$
1	nominal	nominal	nominal	nominal	nominal
2	upper tolerance	nominal	nominal	nominal	nominal
3	lower tolerance	nominal	nominal	nominal	nominal
4	nominal	upper tolerance	nominal	nominal	nominal
5	nominal	lower tolerance	nominal	nominal	nominal
6	nominal	out-of-tolerance	nominal	nominal	nominal
7	nominal	nominal	upper tolerance	nominal	nominal
8	nominal	nominal	lower tolerance	nominal	nominal
9	nominal	nominal	out-of-tolerance	nominal	nominal
10	upper tolerance	upper tolerance	nominal	nominal	nominal
11	lower tolerance	lower tolerance	nominal	nominal	nominal
12	upper tolerance	nominal	upper tolerance	nominal	nominal
13	lower tolerance	nominal	lower tolerance	nominal	nominal
14	nominal	upper tolerance	upper tolerance	nominal	nominal
15	nominal	lower tolerance	lower tolerance	nominal	nominal
16	nominal	out-of-tolerance	out-of-tolerance	nominal	nominal
17	upper tolerance	upper tolerance	upper tolerance	nominal	nominal
18	lower tolerance	lower tolerance	lower tolerance	nominal	nominal
19	nominal	nominal	nominal	upper tolerance	nominal
20	nominal	nominal	nominal	lower tolerance	nominal
21	nominal	nominal	nominal	out-of-tolerance	nominal
22	nominal	nominal	nominal	nominal	upper tolerance
23	nominal	nominal	nominal	nominal	lower tolerance
24	nominal	nominal	nominal	nominal	out-of-tolerance
25	nominal	nominal	nominal	upper tolerance	upper tolerance
26	nominal	nominal	nominal	lower tolerance	lower tolerance
27	nominal	nominal	nominal	out-of-tolerance	out-of-tolerance
28	upper tolerance	upper tolerance	upper tolerance	upper tolerance	upper tolerance
29	lower tolerance	lower tolerance	lower tolerance	lower tolerance	lower tolerance
30	nominal	out-of-tolerance	out-of-tolerance	out-of-tolerance	out-of-tolerance

## Data Availability

The original contributions presented in the study are included in the article, further inquiries can be directed to the corresponding author.

## References

[1] ASM International Handbook Committee (1996). ASM Handbook Volume 19 Fatigue and Fracture.

[2] Rice R.C. (1997). SAE Fatigue Design Handbook.

[3] Wole S., Ferri A., Ferri A.M.H., Winston O.S. (2023). Introduction to Cyclic Loading and Fatigue. Comprehensive Structural Integrity.

[4] Alinejad F., Botto D. Innovative design of attachment for turbine blade rotating at high speed. Proceedings of the ASME Turbo Expo 2017: Turbomachinery Technical Conference and Exposition GT2017.

[5] Khot A., Bharatish A., Srihari P.V. (2017). Effect of fretting fatigue parameters on fir-tree joint of aero-engine blade disc interface. Int. J. Nov. Res. Dev..

[6] Alinejad F., Bessone A., Botto D., Gola M. Design space reduction for the optimization of the blade fir-tree attachment. Proceedings of the ASME Turbo Expo 2018: Turbomachinery Technical Conference and Exposition GT2018.

[7] Botto D., Campagna A., Lavella M., Gola M.M. Experimental and numerical investigation of fretting wear at high temperature for aeronautical alloys. Proceedings of the ASME Turbo Expo 2010: Power for Land, Sea and Air.

[8] Anadavel K., Prakash R.V. (2011). Effect of three-dimensional loading on macroscopic fretting aspects of an aero-engine blade–disc dovetail interface. Tribol. Int..

[9] De Oliveira Vale T., da Costa Villar G., Menezes J.C. (2012). Methodology for Structural Integrity Analysis of Gas Turbine Blades. J. Aerosp. Technol. Manag..

[10] Beisheim J.R., Sinclair G.B. (2008). Three-Dimensional Finite Element Analysis of Dovetail Attachments with and without Crowning. J. Turbomach..

[11] Meguid S.A., Kanth P.S., Czekanski A. (2000). Finite element analysis of fir-tree region in turbine discs. Finite Elem. Anal. Des..

[12] Shankar M., Kumar K., Ajit Prasad S.L. (2010). T-root blades in a steam turbine rotor: A case study. Eng. Fail. Anal..

[13] Acar E., Gündüz M. Structural optimization of blade-disc firtree attachment of an aeroengine. Proceedings of the 16th International Conference on Machine Design and Production.

[14] Song W., Keane A.J. (2004). An efficient evolutionary optimisation framework applied to turbine blade firtree root local profiles. Struct. Multidiscip. Optim..

[15] Rao J.S., Kumar B. 3D blade root shape optimization. Proceedings of the 10th International Conference on Vibrations in Rotating Machinery.

[16] Alinejad F., Botto D. (2019). Innovative adaptive penalty in surrogate-assisted robust optimization of blade attachments. Acta Mech.

[17] Botto D., Lavella M. (2018). Fretting Fatigue Analysis of Additively Manufacture Blade Root Made of Intermetallic Ti-48Al-2Cr-2Nb Alloy at High Temperature. Materials.

[18] Brujic D., Ristic M., Mattone M., Maggiore P., De Poli G.P. (2010). CAD based shape optimization for gas turbine component design. Struct. Multidiscip. Optim..

[19] Moneta G., Jachimowicz J. (2020). Impact of manufactory tolerances on stress in a turbine blade fir-tree root. Fatigue Aircr. Struct..

[20] Xu Y. (2018). Analysis of Barreling and Tolerance Effects of Blade Attachments. Master’s Thesis.

[21] Davis J.R., ASM International Handbook Committee (2000). ASM Specialty Handbook: Nickel, Cobalt, and Their Alloys.

[22] ASM International Handbook Committee (1993). ASM Handbook Volume 1: Properties and Selection: Irons, Steels, and High-Performance Alloys.

[23] (2018). Ansys® Mechanical APDL.

[24] Vergani L. (2001). Meccanica dei Materiali.

[25] Dowling N.E. (2013). Mechanical Behavior of Materials, Methods for Deformation, Fracture, and Fatigue.

[26] Zhu S., Ye W., Correia J.A.F.O., Jesus A.M.P., Wang Q. (2022). Stress gradient effect in metal fatigue: Review and solutions. Theor. Appl. Fract. Mech..

[27] Mei J., Xing S., Vasu A., Chung J., Desai R., Dong P. (2020). The fatigue limit prediction of notched components–A critical review and modified stress gradient based approach. Int. J. Fatigue.

